# Supplementary material for: Gate controlled valley polarizer in bilayer graphene
Source: Nat Commun. 2020 Mar 5;11:1202. doi: 10.1038/s41467-020-15117-y (PMC7058031; doi:10.1038/s41467-020-15117-y)
Supplement: Supplementary file 2 — Supplementary Information [file 41467_2020_15117_MOESM2_ESM.pdf]

# Supplementary Information

# Gate Controlled Valley Polarizer in Bilayer Graphene

Chen et al.

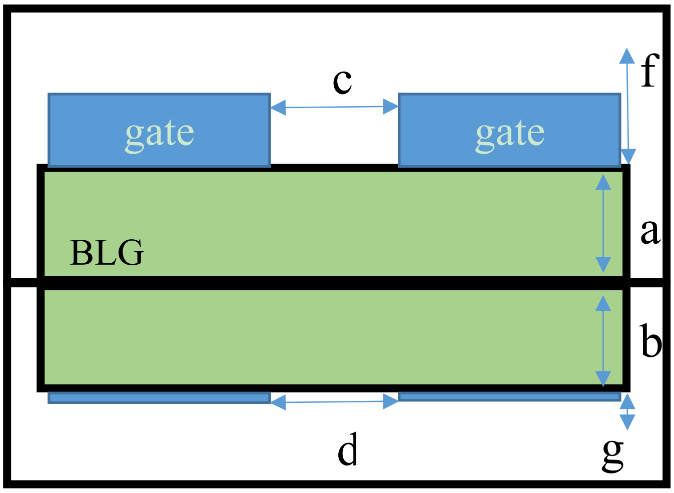


**Supplementary Figure 1** Schematics of the dual split gate structure. Each dimension parameter is denoted near corresponding region.

**Supplementary Note 1: Electrostatic modelling**

MATLAB PDE (partial differential equation) toolbox is a platform which can solve electrostatic distribution, heat transfer, and general partial differential equations (PDEs) using finite element analysis. The dual-split gate pairs are modelled as shown in Supplementary Figure 1.

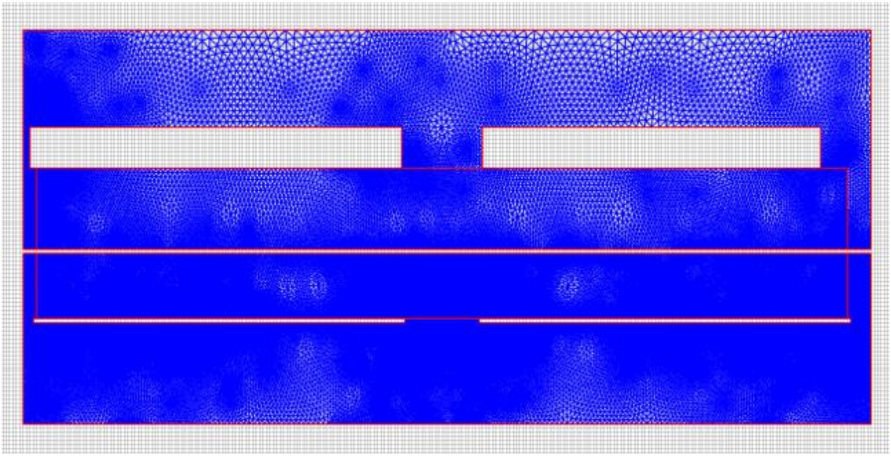

**Supplementary Figure 2** Electrostatic model for dual split gate BLG device. Thickness of top and bottom gates are set to be 25 and 2.5 nm respectively. The potential of BLG and surrounding s are set to be zero when the potential is set to be +/- 5 volts for top/bottom gates.

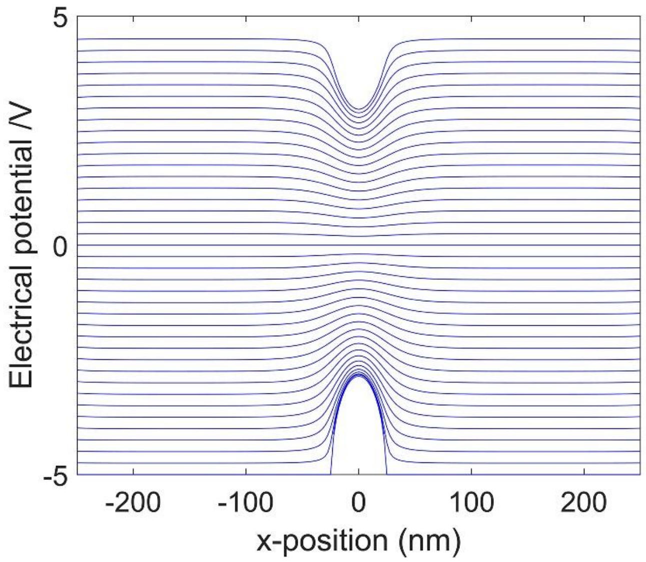


**Supplementary Figure 3** Electrical potential distribution modelled near BLG surface. Curves (from lower to higher) are extracted from z= -62.5 nm to 62.5 nm near BLG surface.

The electrical potentials of boundaries are set and then differential equation $\nabla \cdot D = \rho_f$ is solved. Here different geometries can be tested and optimized to minimize the residual charges in evenly gated configuration.

Supplementary Figure 2 shows the grid to solve the potential distribution within the device. Supplementary Figure 3 shows an example for split gates with same gap, perfect alignment, but different thickness of gate electrodes. As a result, the potential distribution is not perfectly symmetric between top- and bottom gates causing an effective charge accumulation on bilayer graphene in the gapped region.

In order to extract the charge accumulation at bilayer graphene we bias the top- and bottom gates with opposite voltages at voltages of +/- 5 volt. This is about the moderate voltage in our devices before leakage occurs. Below, we discuss two possible scenarios.

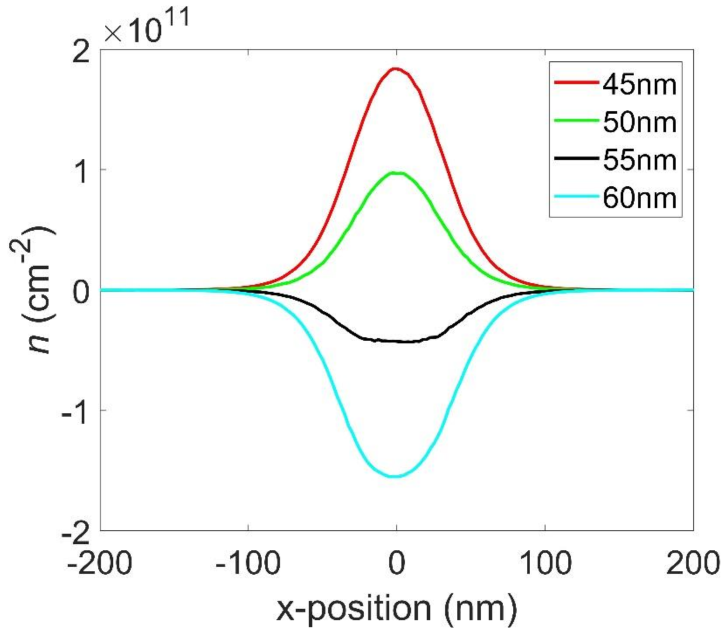


**Supplementary Figure 4** Electron density in x direction modelled with different top gate split gap size when bottom gate split gap is 50nm.

Example 1: Split gap dependence of residual charge.

We consider bottom gates with constant split gap size (50 nm) and dielectric thickness of 50 nm, while the gap size of top gates with dielectric thickness 50 nm is varied. The charge carrier density in BLG is plotted along the z=0 cross-section. Apparently, gap size engineering can reduce the induced density by factor of 5 In this case, top and bottom dielectric layer thickness is both kept at 50nm.

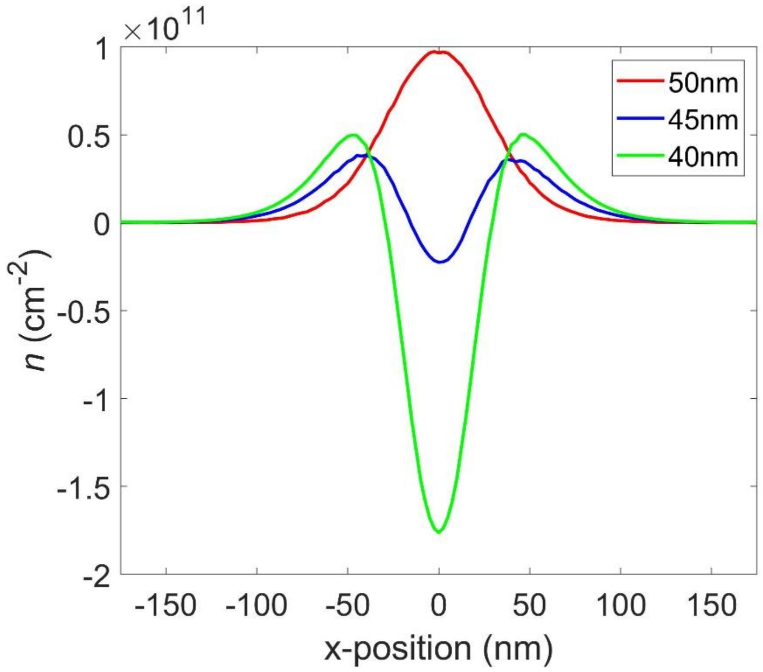


**Supplementary Figure 5** Electron density distribution in x direction modelled with different top dielectric thickness when bottom counterpart is kept 50 nm.

Example 2: Dielectric thickness dependence

Here, we assume as above bottom gates with gap size of 50 nm and dielectric thickness of 50 nm, and top gates a gap of 50nm. Now, we vary the thickness of top dielectric layer while the thickness of bottom one is kept at 50nm. In Supplementary Figure 5 the charge carrier density is plotted against the position with a maximal bias of +/- 5V at the gate electrodes. There exists a clear transition behavior, indicating an optimal parameter.

**Supplementary Note 2: Device fabrication**

**Fabrication of local gates**

Few-layer graphene exfoliated on $SiO_2$/Si wafer will be patterned into bottom gates using electron beam lithography. PMMA 495 A3 resist is used as etch mask. Mechanical cleaning using contact mode AFM is exploited to remove residues on graphene surface. SEM images are subsequently captured to check split gaps. Patterning the graphitic bottom gates demands delicate control of all relevant parameters such as electron beam dose, etch time. Oxygen plasma is used for etching (20 sccm, 16 W). The table below shows some experimental results for optimizing the etching and dose.

| Layers | Dose | Etch time | Results |
|---|---|---|---|
| 3 | 1200 | 10s*1 (April 2_1) | 70nm |
| 2 | 1250 | 10s*5 (March 2019 5_1) | 70nm |
| 3 | 1000 with PEC | 10s*4 (May 6_1) | 65nm |
| 2 | 1000 with PEC | 10s*3 (May 7_1) | 52nm |
| 2 | 1000 with PEC | 10s*3 (July 2019 2_1) | not etched through |
| 2 | 1000 with PEC | 10s*3 (July 2019 2_1) | 50nm |
| 2 | 1000 with PEC | 10s*2 (July 2019 4_1) | not etched through |
| 2 | 1050 | 10s*3 (July 2019 5_2) | 53nm |
| 2 | 1000 | 10s*3 (July 2019 8) | 62nm |

**Supplementary Table 1** Technical details in local bottom gates fabrication.

Subsequently after graphitic bottom gates are fabricated, mechanical cleaning through contact mode in AFM[1] is exploited to clean the resist residual. As shown in Supplementary Figure 6, the cleaned surface of graphene shows roughness of typically 0.12 nm, comparable to that of $SiO_2$ substrate.

SEM images of several samples are demonstrated are shown in Supplementary Figure 7. Gap sizes are well controlled with ±5nm accuracy. Due to the asymmetric geometry of devices, we must alter the top gate gap accordingly. For example, to achieve 50nm top

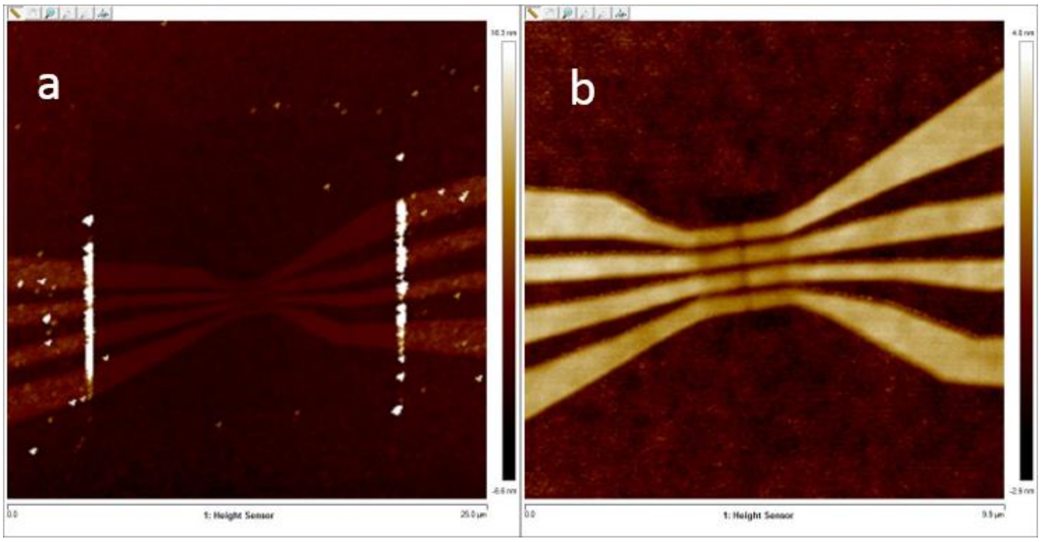


**Supplementary Figure 6** Graphene surface morphology after mechanical cleaning in AFM contact mode. **a** Large scan-range result. **b** Zoom-in view of central region of **a**.

gate gap, the designed pattern for that gap should be 10-15nm larger.

Additionally, the EBL misalignment size can be inferred from Supplementary Figure 7. Second top gate is patterned and deposited with metal after first top gate (two of them share same upper fringe in CAD design). Therefore, the misalignment of EBL process can be estimated from mismatch of upper fringes to be less than 5nm.

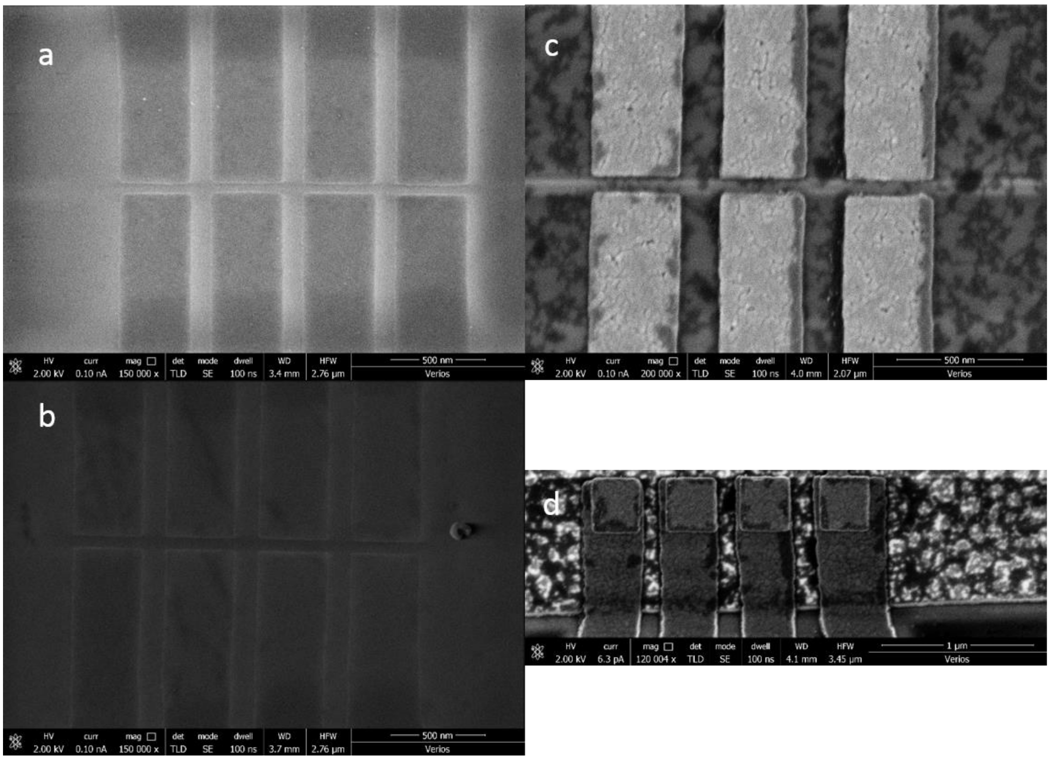


**Supplementary Figure 7** Exemplary SEM images are of bottom gates fabricated using few-layer graphene **a** & **b**, and metal top gates **c** & **d**. Scale bars are shown.

**Stacking BN/BLG/BN heterostructure**

To achieve a homogeneous heterostructure, Van der Waals stacking[2] method is used. Polycarbonate thin film on PDMS scaffold is made and used to pick up hexagonal boron nitride, BLG and hBN layer and then deposited on top of premanufactured graphene bottom gates.

**Supplementary Note 3: Device characterization**

Two terminal measurement of BLG sample is conducted with lock-in amplifier (SRS 830) at frequency of 13.373 Hz. Typically, a small bias $V_{bias}$~100 $\mu$V is applied on the source-drain of the sample and ac current is recorded with lock-in. Keithley 2400 is used for applying gate voltage.

**Mobility and mean free path.**

Devices 1 and 2 are both measured in a cryostat at 1.4K. Electron mobility is extracted in a resistance versus back gate scan (Fig. 2d). Approximated by $R=R_c+(L/W)\cdot(1/ne\mu)$, curve fitting to the two-terminal resistance can be done.

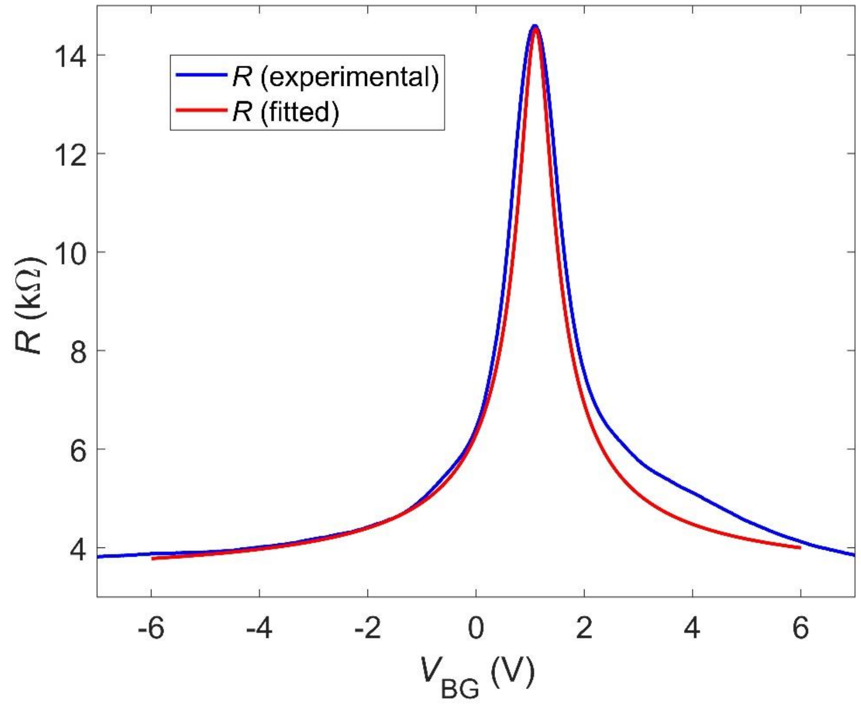


**Supplementary Figure 8** Back-gate dependence of BLG sample resistance. Blue curve is experimental result and red curve is resistance fitted with simulation. Data from device 2.

Simulation is done to fit the broadened resistance peak.

$$n_{eff} = \sqrt{n + n_0}, \qquad n = \frac{CV_{bg}}{e}$$

Here $n_0$ is disorder induced charge in BLG sample and $n$ is gate induced charge. Together $n_{\mathrm{eff}}$ is the effective charge carrier density in BLG. The device geometry is totally 12 μm ×1 μm in rectangular shape. Here $n_0$ used for fitting is ~$2\times10^{10}$ $\mathrm{cm}^2$, consistent with typical residual charge density in encapsulated BLG. Subsequently, summation of contact resistance and resistance from each part can capture most features of experimental result, which justify our fitting process.

The above relation yields carrier mobility of around 150,000 and 320,000 $\mathrm{cm^2\,V^{-1}\,s^{-1}}$ for device 1 and 2 respectively, indicating high sample quality.

$$\mu = \frac{e\tau}{m} = \frac{e}{m}\cdot\frac{L_{mfp}}{v_F}, E_F = \frac{\pi\hbar^2 n}{2m} = \frac{mv_F^2}{2}$$
$$L_{mfp} = \sqrt{\pi n}\cdot\frac{\hbar}{e}\cdot\mu$$

In above relation, electron mobility in Drude model is proportional to mean free path $L_{\mathrm{mfp}}$. Thus, the mean free path is estimated to be 330 and 700 nm for device 1 and 2, respectively. We find that for our devices the mean free path is comparable to the gate length (~400 nm), indicating ballistic transport in the channel.

**Calculation of *n* and *D*-field**

In dual-gate scan, e.g., when voltage potentials on top and bottom gates are varied, the charge carrier density $n$ and displacement field $D$-field will be modified accordingly. The relation of them to top/bottom gate voltages can be derived as

$$n= \varepsilon_0\varepsilon_r((V_t-V_{t0})/d_t+(V_b-V_{b0}/d_b))/e,$$

$$D= \varepsilon_r((V_t-V_{t0})/d_t -(V_b-V_{b0}/d_b))/2.$$

Here, $\varepsilon_0$, $\varepsilon_r$, $V_t$, $V_{t0}$, $V_b$, $V_{b0}$, $d_t$, $d_b$, $e$, is vacuum electric constant, dielectric constant, top-gate voltage, top-gate voltage offset, bottom gate voltage, bottom gate voltage offset, top/bottom dielectric thickness and elementary charge. The $\varepsilon_r$ for hBN here is taken as 4 as used in previous experimental work[3]. The dielectric thickness for top and bottom hBN layer is measured from atomic force microscopy. Voltage offsets for top/bottom gates originate from disorder induced doping, either from lithography or hetero-structure inhomogeneity. Here $V_{t0}$ ~ 0.25 V, $V_{b0}$ ~0.5 V for Fig. 3b and $V_{t0}$ ~ 0.05 V, $V_{b0}$ ~0.5V for Fig. 4b, which is consistent with a slightly doped regime.

**Supplementary Note 4: Supplementary data from device 1 and device 2**

Since the gate-pair setup is quadrupole-like, mirror images in the gate-gate scans are expected when swapping the gate configurations in up-down or left-right symmetry. Specifically, in our measurements, and as denoted in insets of Fig. 3b&d, the left half pair gates are at constant potentials (left half- pair: TG+/BG-). As a result of swapping the polarization of fixed potential top- and bottom gates (changing the up-down symmetry: left half- pair: TG-/BG+), a mirror image appears in the gate-gate scan with the insulating region appearing now on the left. To further illustrate the controllable valley polarization, the left-right mirror image results are shown in Supplementary Figure 9. Here the gate configurations are that the right half-pair is at fixed potential (Supplementary Figure 9a): TG+/BG- and (Supplementary Figure 9b): TG-/BG+. The mirror image results also show plateaus/linearly increasing features in log-view resistance under odd/even field configurations, consistent with previous observation. The on/off ratios in this mirror image result for device 1 are listed in Supplementary Table 2.

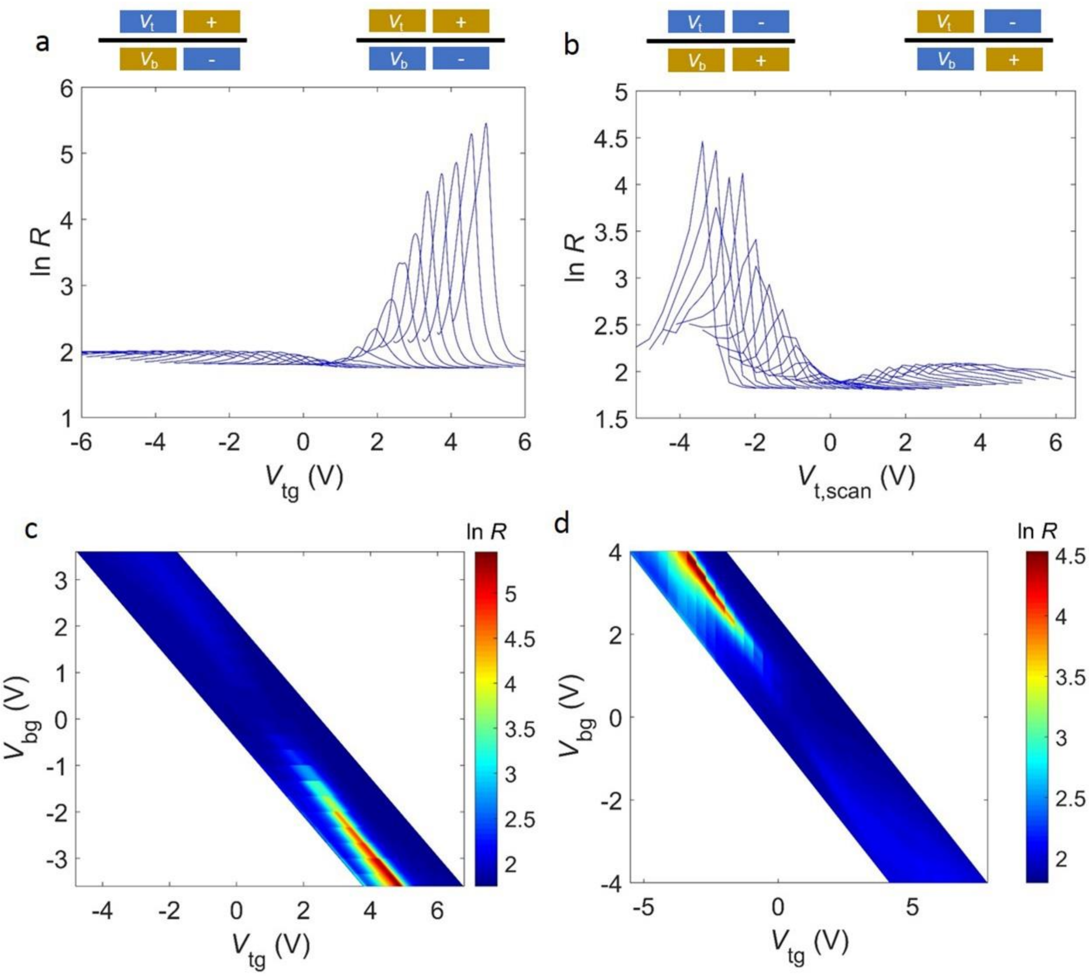


**Supplementary Figure 9** Supplementary data from device 1. The right half-pair is fixed at constant potential, as mirror (left-right) image result of Fig. 3. **a** Resistance (in log-scale) as function of top gate potential ($V_{tg}$) at different bottom gate potentials ($V_{bg}$). **b** Color-plot of resistance (in log-scale) as function of top and bottom gate potential. **c** Resistance as function of top gate potential at different bottom gate potentials (Opposite fixed gate polarity, as denoted in insets of **a** and **c**). Inset is zoom-in view of sample resistance, where left region corresponds to pp' junction and right region corresponds to pn junction condition.  **d**, Color-plot of resistance as function of top and bottom gate potential. Insets in (a)&(b) are schematics of gate configurations.

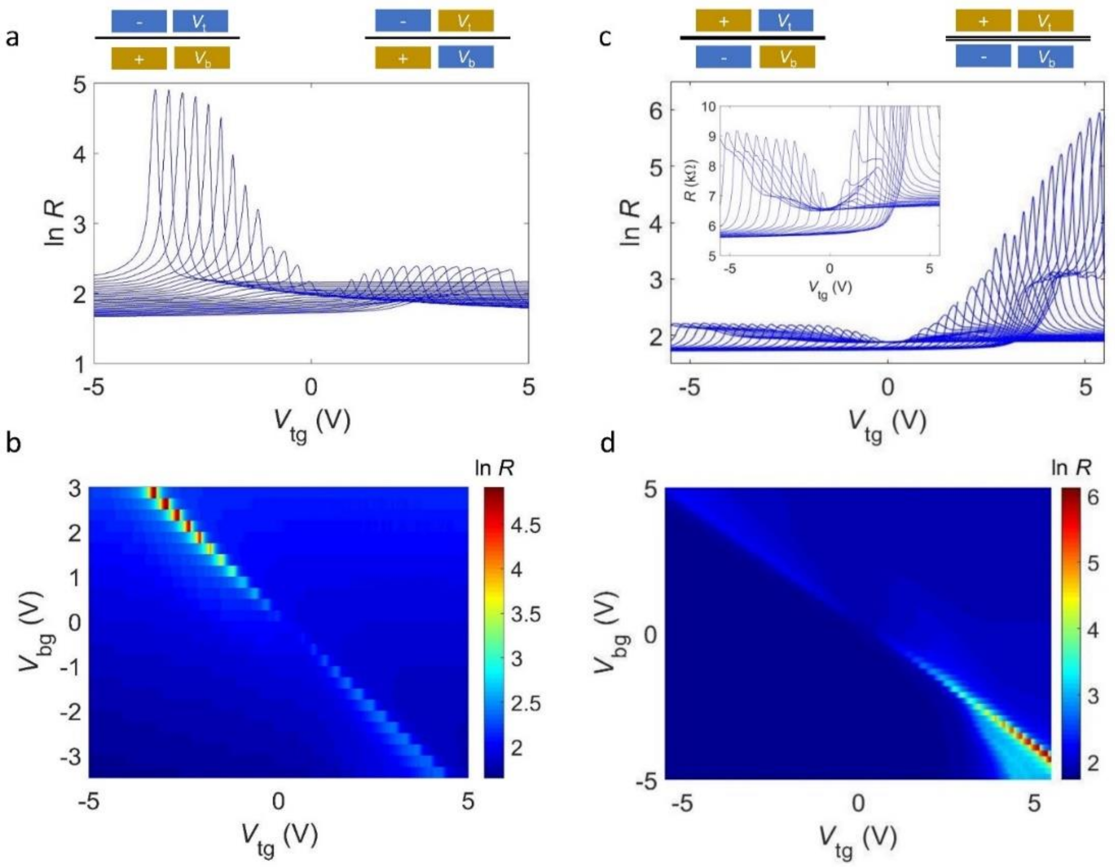


**Supplementary Figure 10** Data from device 2. **a** Resistance (in log-scale) as function of top gate potential ($V_{tg}$) at different bottom gate potentials ($V_{bg}$). **b** Color-plot of resistance (in log-scale) as function of top and bottom gate potential. **c** Resistance as function of top gate potential at different bottom gate potentials (Opposite fixed gate polarity, as denoted in insets of **a** and **c**). Inset is zoom-in view of sample resistance, where left region corresponds to pp' junction and right region corresponds to pn junction condition. **d**, Color-plot of resistance as function of top and bottom gate potential. Insets are schematics of gate configurations.

Additional data from device 2 also provides consistent result in our valley polarizer regime. Supplementary Figure 10a and b show dual-gate scan result when half of the BLG is kept at positive E-field polarity, similar to that in Fig. 3. Supplementary Figure 10c & d show dual-gate scan results with opposite E-field (negative polarity) with respect to Supplementary

Figure 10a & b. The on/off ratios in each configuration for device 2 are also listed in Supplementary Table 2.

**Supplementary Note 5: Extraction of chiral channel resistance**

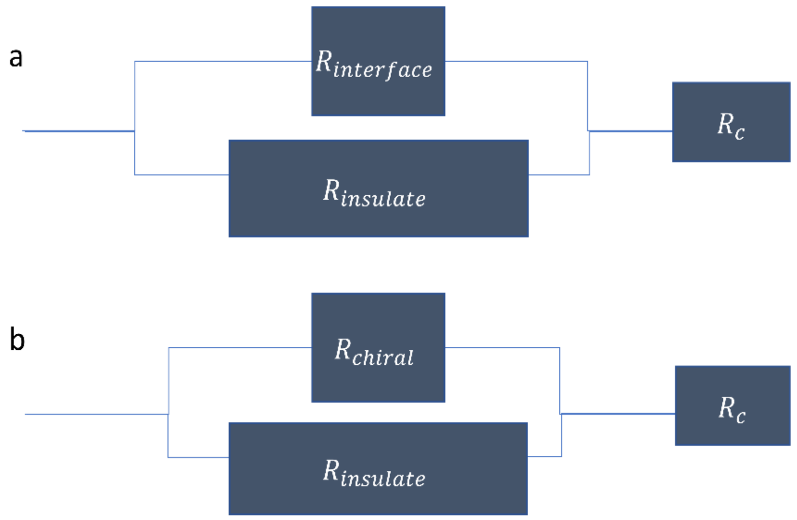


**Supplementary Figure 11** Schematics model of contact resistance, chiral channel resistance in chiral channel configuration. **a** At high densities, there could exist interface resistance due to pn or pp' junction in parallel with fixed insulating region resistance. **b** Near charge neutral point, the chiral channel in parallel with resistance constitutes to the measured resistance.

A multiple-resistor model shown in Supplementary Figure 11 is used to showcase the different components. In this model, in the chiral channel configuration there exist chiral channel resistance $R_{\text{chiral}}$ at charge neutral point (b) and interface resistance $R_{\text{interface}}$ (a) which originates from pn or pp' junction resistance when locally gated region is at high density. Note, contact resistance in this context comprises series resistance of non-gated regions of BLG and metal to sample contact resistance.

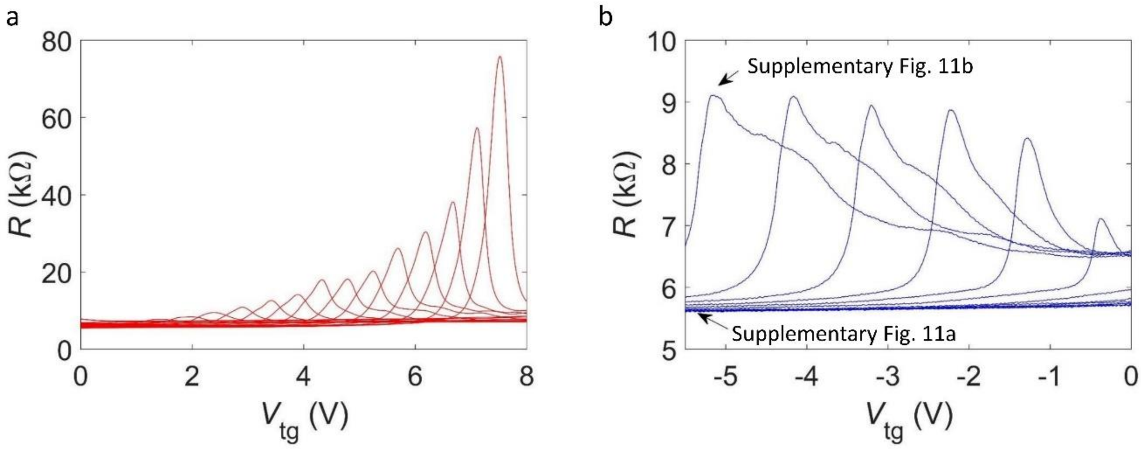


**Supplementary Figure 12** BLG resistance as function of top gate potential at fixed bottom gate potentials. **a** (insulating regime): at high densities, the saturated base is taken as $R_c$. **b** (chiral channel regime): at high density, the saturated base resistance (corresponds to Supplementary Figure 11a) consists of interface resistance while near charge neutral point (corresponds to Supplementary Figure 11b) only chiral states contributes to resistance.

For instance, the contact resistance in Fig. 3c is extracted from baseline in Supplementary Figure 12a. Note that fixed gate voltages in Supplementary Figure 12a (half of the gates at TG/BG (9V/-7V)) is slightly different from Fig. 3c (TG/BG (8.5V/-7V)), due to slightly hysteric behavior in locating BLG at charge neutral point.

We find that in p- and n- doped side of the curves in Supplementary Figure 10a and c, base resistances are different. The inset of Supplementary Figure 10c shows a zoom-in view of sample resistance. Since the sample itself is slightly p- doped (easily seen from Supplementary Figure 8), there could be an interface resistance when the gated region is at high density (pp' junction or pn junction). Although the exact value of these junctions cannot be determined from our 2-terminal data, we observe that the difference of resistances between pp' and pn junctions can reach ~1 kOhm. This may indicate that the resistance at high gate voltages does not only consist of the series resistance, but also includes

contributions of pn-junction resistance of several kOhm. This pn-junction resistance could explain the mismatch between expected and experimentally extracted channel resistance.

**Supplementary Table 2** Table of extracted resistances.

| Device source | $R_c$ | $R_{insulating}$ | $R_{chiral}$ | Contrast |
|---|---|---|---|---|
| Device 1 in Fig. 3 | 5.48 | 553.5 | 5.72 | ~96 |
| Device 1 in Fig. 4 | 5.68 | 181.0 | 1.75 | ~103 |
| Device 1 in Supplementary Figure 9 | 5.5 | 234 | 1.97 | ~110 |
| Device 1 in Supplementary Figure 9 | 6.03 | 86 | 2.37 | ~36 |
| Device 2 in 'positive' polarity | 5.61 | 448.7 | 3.53 | ~126 |
| Device 2 in 'negative' polarity | 5.21 | 130.9 | 5.88 | ~22 |
| Device in ref. [4] (at 0T) | N.A. | ~100 | ~10 | ~10 |
| Device in ref. [5] (at 0T) | 0.8 | 19.3 | 6.45 | <5 |
| Device in ref. [5] (at 2T) | N.A. | ~36 | 6.45 | ~6 |
| Device in ref. [5] (at 4T) | N.A. | ~210 | 6.45 | ~30 |
| Device in ref. [5] (at 5T) | N.A. | ~1200 | 6.45 | ~180 |

Supplementary Table 2 shows critical data of devices in this work and previous works [4,5]. Note resistance values are in kOhm unit.

**Supplementary Note 6: Valley analyzer measurement**

As shown in Fig. 5 in main text, the valley analyzer consists of an additional two gate-pairs (each comprises of four gates) in series to the first valley-polarizer pair. Also, the gate electrodes of the analyzer can be individually configured to generate same or opposite valley polarization.

In practice, the 1st gate-pair (the left one in device schematics) is applied with constant voltages (pre-determined from above measurements) to remain in charge neutral valley polarized state (e.g., point b in Supplementary 12) throughout all measurements to be conducted with the analyzer. Then the gate potentials for the analyzer are varied with the method shown in Fig. 3&4, that is, a half gate-pair is kept fixed at charge neutral point, for the other half we conduct a gate-gate-scan for the remaining top- and bottom gate.

The data of Fig. 5 are thus acquired from two measurements: one of them is measured with half gate-pair at positive *D*-field, the other one at negative *D*-field. The reason why we do not combine two diagonal gates in 2nd pair to measure the above two state in one measurement is that charge neutrality points in upper and lower parts are not always the same.

Valley indexed channels are shown in the two-contrasting state in the schematics. In principle, the topological channels cannot mix with each other except for large momentum transfer, which should allow observable resistance difference in electrical measurement.

As a control, we also measured the resistance with the second gate in the insulating state (Supplementary Figure 13). We observe similar resistances to the valley blocked configuration (b), although not as highly insulating states as in the sole polarizer configuration are reached. This may have its cause in device imperfections, i.e. different charge neutral point for neighboring gate pairs.

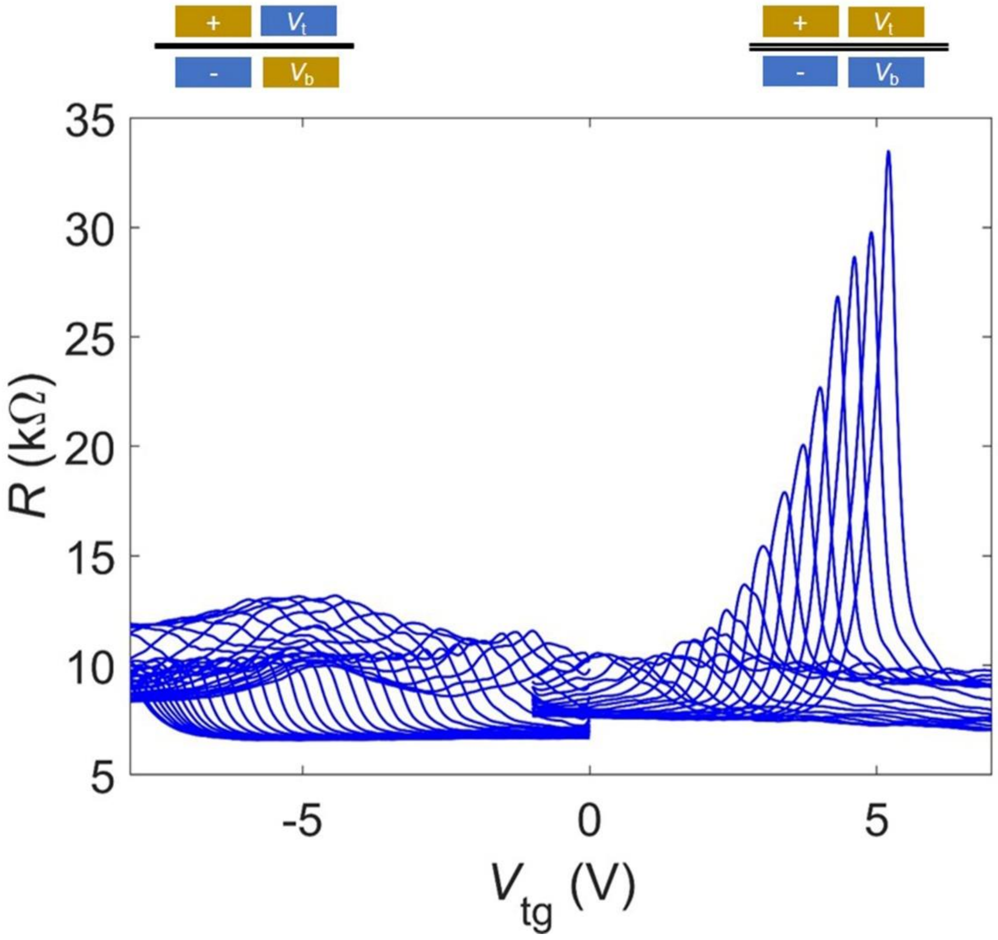


**Supplementary Figure 13**. Individual measurement of 2nd gate-pair when half-pair is fixed at constant voltages to remain charge neutral. The left part is the result acquired for Fig. 5 (polarizer state). The right part is the normal field-induced insulating state. Corresponding gate configurations are shown with schematics above.

## Supplementary references


1 Goossens, A. M. *et al.* Mechanical cleaning of graphene. *Applied Physics Letters* **100**, 073110, doi:10.1063/1.3685504 (2012).

2 Zomer, P. J., Guimarães, M. H. D., Brant, J. C., Tombros, N. & van Wees, B. J. Fast pick up technique for high quality heterostructures of bilayer graphene and hexagonal boron nitride. *Applied Physics Letters* **105**, 013101, doi:10.1063/1.4886096 (2014).

3 Dean, C. R. *et al.* Boron nitride substrates for high-quality graphene electronics. *Nature Nanotechnology* **5**, 722-726, doi:10.1038/nnano.2010.172 (2010).

4 Li, J. *et al.* Gate-controlled topological conducting channels in bilayer graphene. *Nature Nanotechnology* **11**, 1060, doi:10.1038/nnano.2016.158

https://www.nature.com/articles/nnano.2016.158#supplementary-information (2016).

5 Li, J. *et al.* A valley valve and electron beam splitter. *Science* **362**, 1149, doi:10.1126/science.aao5989 (2018).
